# Increase in Homeostasis Model Assessment of Insulin Resistance (HOMA-IR) Had a Strong Impact on the Development of Type 2 Diabetes in Japanese Individuals with Impaired Insulin Secretion: The Saku Study

**DOI:** 10.1371/journal.pone.0105827

**Published:** 2014-08-28

**Authors:** Akiko Morimoto, Yukako Tatsumi, Fumie Soyano, Naomi Miyamatsu, Nao Sonoda, Kayo Godai, Yuko Ohno, Mitsuhiko Noda, Kijyo Deura

**Affiliations:** 1 Department of Clinical Nursing, Shiga University of Medical Science, Otsu, Shiga, Japan; 2 Department of Mathematical Health Science, Graduate School of Medicine, Osaka University, Suita, Osaka, Japan; 3 Saku Central Hospital, Saku, Nagano, Japan; 4 Department of Diabetes Research, National Center for Global Health and Medicine, Shinjuku-ku, Tokyo, Japan; Virgen Macarena University Hospital, School of Medicine, University of Seville, Spain

## Abstract

Our aim was to assess the impact of increase in homeostasis model assessment of insulin resistance (HOMA-IR) on the development of type 2 diabetes in Japanese individuals with impaired insulin secretion (IIS). This study included 2,209 participants aged 30–69 without diabetes at baseline who underwent comprehensive medical check-ups between April 2006 and March 2007 at Saku Central Hospital. Participants were classified into eight groups according to the combination of baseline IIS status (non-IIS and IIS) and category of HOMA-IR change between the baseline and follow-up examinations (decrease, no change/small increase, moderate increase, and large increase). Type 2 diabetes was determined from fasting and 2 h post-load plasma glucose concentrations at the follow-up examination between April 2009 and March 2011. At baseline, 669 individuals (30.3%) were classified as having IIS. At follow-up, 74 individuals developed type 2 diabetes. After adjusting for confounding factors including baseline HOMA-IR values, the multivariable-adjusted odds ratios (95% confidence intervals) for type 2 diabetes in the non-IIS with a decrease (mean change in HOMA-IR: −0.47), non-IIS with a moderate increase (mean change in HOMA-IR: 0.28), non-IIS with a large increase (mean change in HOMA-IR: 0.83), IIS with a decrease (mean change in HOMA-IR: −0.36), IIS with no change/small increase (mean change in HOMA-IR: 0.08), IIS with a moderate increase (mean change in HOMA-IR: 0.27), and IIS with a large increase (mean change in HOMA-IR: 0.73) groups, relative to the non-IIS with no change/small increase (mean change in HOMA-IR: 0.08) group were 0.23 (0.04, 1.11), 1.22 (0.26, 5.72), 2.01 (0.70, 6.46), 1.37 (0.32, 4.28), 3.60 (0.83, 15.57), 5.24 (1.34, 20.52), and 7.01 (1.75, 24.18), respectively. Moderate and large increases in HOMA-IR had a strong impact on the development of type 2 diabetes among individuals with IIS in this Japanese population.

## Introduction

The incidence of type 2 diabetes is significantly increasing in Asian countries [1]. Furthermore, Asian Americans have been found to be at significantly higher risk of type 2 diabetes than whites, despite having substantially lower body mass index (BMI) [2]. Impaired insulin secretion (IIS) and insulin resistance (IR) are the main pathophysiological components of type 2 diabetes [3]–[5], with the contributions of these factors thought to differ between Asians and whites.

We recently reported on the impact of IIS and IR on the incidence of type 2 diabetes in a Japanese population [6], [7]. In that study, IIS had a great impact on the incidence of type 2 diabetes in Japanese individuals [6], [7]. It is therefore important to clarify the mechanisms that lead to the development of type 2 diabetes in individuals with IIS. It is thought that those with IIS cannot compensate for an increase in IR compared with those without IIS. The objective of this study was to assess the impact of increase in homeostasis model assessment of insulin resistance (HOMA-IR) on the development of type 2 diabetes in Japanese individuals with IIS.

## Materials and Methods

### Study population

The Saku study included community residents who underwent comprehensive medical check-ups for the prevention and early detection of various diseases, including diabetes, cardiovascular disease and cancer, at Saku Central Hospital, one of the core hospitals in the Nagano prefecture, located in Saku city, Japan. The full details of this study have been previously described [6]. The cohort consisted of 4,318 individuals, aged 30–69 years, who underwent a baseline comprehensive medical check-up over 2 days and 1 night between April 2006 and March 2007 at Saku Central Hospital. Of these, 3,758 did not have diabetes at baseline, based on three criteria: (1) no history of diabetes, as determined by interviews conducted by the physicians; (2) fasting plasma glucose (FPG) concentration <7.0 mmol/l; and (3) 2 h post-load plasma glucose (PG) concentration <11.1 mmol/l. Of these 3,758 individuals, 2,671 (71.1%) underwent a follow-up comprehensive medical check-up over 2 days and 1 night between April 2009 and March 2011. Because IR is liable to change as a result of diseases or interventions, we excluded 436 individuals with a history of cancer and/or cardiovascular disease at the follow-up examination and eight individuals who received medical treatment for diabetes between the baseline and follow-up examinations. Additionally, we excluded 18 individuals with missing data at the baseline and/or follow-up examinations. Thus, a total of 2,209 individuals (1,225 men and 984 women), aged 30–69 years, were eligible for our analysis.

### Ethics Statement

The study protocol was in accordance with the Declaration of Helsinki and was approved by the Ethics Committee of Saku Central Hospital. Written informed consent was obtained from each participant.

### Procedures

All participants were fasted overnight (12 h), and had a standard 75 g oral glucose tolerance tests (OGTTs) in the morning. Blood samples were obtained at 0 (fasting), 30, 60 and 120 min, with PG measured on all four occasions and serum insulin concentrations measured at 0 and 30 min in the clinical laboratory of Saku Central Hospital. Serum insulin concentrations were measured using a chemiluminescence enzyme immunoassay (Lumipulse Presto Insulin, Fujirebio Inc., Tokyo, Japan). Blood glucose, high density lipoprotein cholesterol, and serum triacylglycerol concentrations were measured by enzymatic methods. Weight, height, waist circumference and body fat percentage were measured in the morning during the fasting state. BMI was calculated as the weight (kg) divided by the height squared (m^2^). Waist circumference was measured around the abdomen at the level of the navel at the late expiratory phase using a tape measure. Body fat percentage was evaluated by the bioelectric impedance method with an automatic scale. Blood pressure was measured by trained nurses using an automatic sphygmomanometer, with the individual in the sitting position after resting for at least 5 min. Each check-up also included standard questionnaires on demographic characteristics, medical history, family history and health-related habits. Alcohol consumption (ethanol) was categorised as 0 g/week, 1–139 g/week, or ≥140 g/week, and exercise was categorised as 0 min/week, 1–119 min/week, or ≥120 min/week.

### Definition of IIS and category of HOMA-IR change

Figure S1 in File S1 shows definition of IIS and category of HOMA-IR change. The insulinogenic index was calculated using the formula: insulinogenic index = (Insulin_30_ [pmol/l]−Insulin_0_ [pmol/l])/(Glucose_30_ [mmol/l]−Glucose_0_ [mmol/l]) [8]. We defined IIS as an insulinogenic index ≤51.7 pmol/mmol (40.0 µU/mg) [6], [9], [10]. HOMA-IR was calculated using the formula: HOMA-IR = Insulin_0_ (µU/ml) Glucose_0_ (mmol/l)/22.5 [11]. HOMA-IR change between the baseline and follow-up examinations (ΔHOMA-IR) was calculated using the formula: ΔHOMA-IR = HOMA-IR value at follow-up examination − HOMA-IR value at baseline examination. First, participants were classified into two groups according to ΔHOMA-IR; those with a decrease in HOMA-IR (<0) and those with no change or an increase in HOMA-IR (≥0). Additionally, those with no change or an increase in HOMA-IR (≥0) were divided into tertiles; tertile 1, those with no change or a small increase (0.00–0.16); tertile 2, those with a moderate increase (0.17–0.40); and tertile 3, those with a large increase (≥0.41) in HOMA-IR. Participants in the study were classified into eight groups according to the combination of baseline IIS status (non-IIS and IIS) and category of ΔHOMA-IR (decrease, no change/small increase, moderate increase, and large increase).

### Definition of type 2 diabetes

Type 2 diabetes was determined using the 1999 World Health Organization criteria [12] (FPG ≥7.0 mmol/l and/or 2 h post-load PG ≥11.1 mmol/l) at the follow-up examination between April 2009 and March 2011.

### Statistical analysis

Differences in baseline characteristics among the eight groups were determined by: analysis of covariance with adjustments for age and sex for normally distributed continuous data; Kruskal-Wallis H tests for non-normally distributed continuous data; and *χ* ^2^ tests for dichotomous and categorical data.

Logistic regression analysis was used to estimate the multivariable-adjusted odds ratios (ORs) and 95% confidence intervals (CIs) for the development of type 2 diabetes among the eight groups. The non-IIS with no change/small increase in HOMA-IR group was used as the reference group. Age, sex, and follow-up years (3 or 4 years) were included in model 1; and all factors in model 1 plus family history of diabetes (yes or no), current smoking (yes or no), alcohol consumption (0 g/week, 1–139 g/week or ≥140 g/week), exercise (0 min/week, 1–119 min/week or ≥120 min/week), baseline HOMA-IR, FPG, and 2 h PG were included in model 2. Moreover, we added eight individuals who received medical treatment for diabetes between the baseline and follow-up examinations. In these 2,217 individuals, data were adjusted for all factors in model 2.

In order to assess the impact of severe IIS and HOMA-IR increases on the development of type 2 diabetes, baseline IIS status was classified into stage 1 (insulinogenic index 35.5–51.7 pmol/mmol) and stage 2 (insulinogenic index 2.8–35.4 pmol/mmol) using the median of the insulinogenic index (35.5 pmol/mmol). Participants were classified into 12 groups according to the combination of baseline IIS status (non-IIS, stage 1 IIS, and stage 2 IIS) and category of ΔHOMA-IR (decrease, no change/small increase, moderate increase, and large increase). Logistic regression analysis was used to estimate the multivariable-adjusted ORs and 95% CIs for the development of type 2 diabetes among these 12 groups. The non-IIS with no change/small increase in HOMA-IR group was used as the reference group. Data were adjusted for age, sex, follow-up years, family history of diabetes, current smoking, alcohol consumption, exercise, baseline HOMA-IR, FPG, and 2 h PG.

Additionally, in order to confirm the risk factors for an increase in HOMA-IR in the non-IIS and IIS groups, multivariable-adjusted ORs and 95% CIs for moderate and large increases in HOMA-IR according to major risk factors for diabetes were calculated using multinomial logistic regression analysis in the non-IIS and IIS groups.

All data were analysed using SPSS statistical software (version 21.0J; SPSS Japan, Tokyo, Japan). All reported *p* values are two-tailed; values <0.05 were considered statistically significant.

## Results

### Characteristics

The mean age of the 2,209 participants was 55.5 years, mean BMI 23.1 kg/m^2^, mean HOMA-IR 1.16 (1.26 in the non-IIS group and 0.91 in the IIS group), and median insulinogenic index 74.9 pmol/mmol (99.7 pmol/mmol in the non-IIS group and 35.5 pmol/mmol in the IIS group). At baseline, 669 individuals (30.3%) were classified as having IIS. Table 1 shows the characteristics of the eight groups. Age- and sex-adjusted mean ΔHOMA-IR in the non-IIS with a decrease in HOMA-IR, non-IIS with no change/small increase in HOMA-IR, non-IIS with a moderate increase in HOMA-IR, non-IIS with a large increase in HOMA-IR, IIS with a decrease in HOMA-IR, IIS with no change/small increase in HOMA-IR, IIS with a moderate increase in HOMA-IR, and IIS with a large increase in HOMA-IR groups were −0.47, 0.08, 0.28, 0.83, −0.36, 0.08, 0.27, and 0.73, respectively. All variables at baseline, except for family history of diabetes, exercise, and systolic blood pressure, differed significantly among the eight groups.

**Table 1:** Characteristics according to baseline IIS status and category of ΔHOMA-IR.

Characteristic	Non-IIS			IIS					*p* value
	Category of ΔHOMA-IR			Category of ΔHOMA-IR					
	Decrease (*n* = 817)	No change/Small increase (*n* = 236)	Moderate increase (*n* = 227)	Large increase (*n* = 260)	Decrease (*n* = 300)	No change/Small increase (*n* = 128)	Moderate increase (*n* = 137)	Large increase (*n* = 104)	
Insulinogenic index (pmol/mmol)	103.4 (74.1, 168.6)	93.6 (69.0, 146.2)	92.0 (65.1, 135.7)	107.3 (74.3, 165.5)	35.3 (23.0, 44.2)	35.1 (25.7, 43.3)	34.8 (26.8, 43.7)	37.8 (27.0, 45.5)	
ΔHOMA-IR	−0.47 (−0.50, −0.44)	0.08 (0.04, 0.12)	0.28 (0.23, 0.34)	0.83 (0.77, 0.88)	−0.36 (−0.40, −0.31)	0.08 (0.04, 0.12)	0.27 (0.20, 0.34)	0.73 (0.64, 0.81)	
Baseline HOMA-IR	1.47 (1.42, 1.52)	0.93 (0.83, 1.02)	0.90 (0.80, 0.99)	1.26 (1.17, 1.35)	1.10 (1.01, 1.18)	0.70 (0.58, 0.83)	0.66 (0.54, 0.78)	0.89 (0.75, 1.04)	<0.001
Age (years)	55.4 (54.9, 55.9)	55.5 (54.6, 56.5)	55.4 (54.4, 56.4)	54.3 (53.4, 55.2)	56.5 (55.7, 57.4)	56.1 (54.8, 57.5)	56.2 (54.9, 57.5)	55.2 (53.7, 56.7)	0.047
Men, n (%)	412 (50)	132 (56)	119 (52)	146 (56)	177 (59)	82 (64)	94 (69)	63 (61)	0.001
Family history of diabetes, n (%)	146 (18)	39 (17)	41 (18)	55 (21)	60 (20)	30 (23)	29 (21)	25 (24)	0.499
Current smoker, n (%)	110 (14)	34 (14)	36 (16)	46 (18)	64 (21)	34 (27)	39 (29)	33 (32)	<0.001
Alcohol consumption (ethanol), n (%)									0.002
0 g/week	304 (37)	87 (37)	86 (38)	88 (34)	86 (29)	36 (28)	34 (25)	36 (35)	
1–139 g/week	344 (42)	86 (36)	100 (44)	116 (45)	128 (43)	56 (44)	54 (39)	45 (43)	
≥140 g/week	169 (21)	63 (27)	41 (18)	56 (22)	86 (29)	36 (28)	49 (36)	23 (22)	
Exercise, n (%)									0.358
0 min/week	383 (47)	115 (49)	101 (45)	133 (51)	134 (45)	58 (45)	64 (47)	49 (47)	
1–119 min/week	283 (35)	68 (29)	69 (30)	80 (31)	101 (34)	46 (36)	40 (29)	27 (26)	
≥120 min/week	151 (19)	53 (23)	57 (25)	47 (18)	65 (22)	24 (19)	33 (24)	28 (27)	
BMI (kg/m^2^)	23.5 (23.3, 23.7)	22.8 (22.4, 23.2)	22.5 (22.1, 22.9)	24.2 (23.9, 24.6)	22.6 (22.3, 22.9)	22.1 (21.6, 22.5)	22.1 (21.6, 22.5)	23.6 (23.1, 24.1)	<0.001
Obesity (BMI ≥25 kg/m^2^), n (%)	239 (29)	49 (21)	37 (16)	100 (39)	49 (16)	17 (13)	19 (14)	30 (29)	<0.001
Waist circumference (cm)	84.4 (83.8, 84.9)	82.3 (81.3, 83.3)	82.0 (81.0, 83.0)	86.0 (85.0, 86.9)	82.0 (81.1, 82.8)	80.0 (78.7, 81.4)	80.9 (79.6, 82.2)	84.7 (83.2, 86.2)	<0.001
Body fat (%)	25.8 (25.4, 26.2)	24.6 (23.9, 25.3)	24.4 (23.7, 25.1)	26.9 (26.3, 27.6)	24.1 (23.5, 24.7)	23.0 (22.1, 23.9)	23.5 (22.6, 24.4)	25.8 (24.8, 26.8)	<0.001
Systolic blood pressure (mmHg)	119.9 (118.8, 120.9)	119.8 (117.8, 121.8)	118.8 (116.8, 120.8)	119.7 (117.9, 121.6)	118.3 (116.5, 120.0)	118.5 (115.8, 121.1)	119.2 (116.6, 121.8)	120.8 (117.8, 123.7)	0.741
Diastolic blood pressure (mmHg)	74.2 (73.5, 75.0)	73.7 (72.4, 75.1)	73.5 (72.1, 74.9)	74.4 (73.1, 75.7)	72.2 (71.0, 73.4)	71.2 (69.3, 73.0)	73.1 (71.3, 74.8)	73.6 (71.6, 75.7)	0.019
HDL-cholesterol (mmol/l)	1.46 (1.44, 1.49)	1.54 (1.49, 1.58)	1.48 (1.44, 1.53)	1.42 (1.38, 1.46)	1.58 (1.54, 1.62)	1.54 (1.48, 1.60)	1.66 (1.60, 1.72)	1.53 (1.46, 1.60)	<0.001
Triacylglycerol (mmol/l)	1.10 (0.80, 1.60)	1.00 (0.70, 1.40)	1.00 (0.70, 1.30)	1.10 (0.80, 1.70)	1.00 (0.70, 1.40)	0.90 (0.70, 1.40)	0.90 (0.60, 1.30)	1.10 (0.80, 1.40)	<0.001
Fasting plasma glucose (mmol/l)	5.51 (5.48, 5.54)	5.35 (5.30, 5.41)	5.36 (5.31, 5.42)	5.40 (5.34, 5.45)	5.63 (5.58, 5.67)	5.35 (5.27, 5.42)	5.31 (5.24, 5.39)	5.45 (5.37, 5.54)	<0.001
2 h post-load plasma glucose (mmol/l)	6.48 (6.39, 6.57)	6.32 (6.16, 6.49)	6.37 (6.20, 6.54)	6.39 (6.23, 6.54)	6.75 (6.60, 6.89)	6.49 (6.26, 6.71)	6.68 (6.46, 6.89)	6.94 (6.69, 7.19)	<0.001
Pre-diabetes, n (%)	177 (22)	33 (14)	40 (18)	47 (18)	103 (34)	26 (20)	37 (27)	37 (36)	<0.001

IIS, impaired insulin secretion; HOMA-IR, homeostasis model assessment of insulin resistance; BMI, body mass index; HDL, high density lipoprotein.

Non-IIS, insulinogenic index >51.7 pmol/mmol (40.0 µU/mg); IIS, insulinogenic index ≤51.7 pmol/mmol (40.0 µU/mg).

A decrease group consisted of individuals with a decrease (<0) in HOMA-IR, whereas those with no change or an increase in HOMA-IR were divided into tertiles; a no change/small increase group, tertile 1 (0.00–0.16); a moderate increase group, tertile 2 (0.17–0.40); and a large increase group, tertile 3 (≥0.41).

Δ = follow-up examination minus baseline examination.

Pre-diabetes, 6.1 mmol/l ≤ fasting plasma glucose <7.0 mmol/l and/or 7.8 mmol/l ≤2 h post-load plasma glucose <11.1 mmol/l.

Dichotomous and categorical data were analyzed by χ^2^ test, and are shown as number (%). Continuous, normally distributed data were analyzed by analysis of covariance with adjustments for age and sex, and are shown as age- and sex-adjusted mean (95% confidence interval). Continuous, non-normally distributed data (insulinogenic index and triacylglycerol) were analyzed by Kruskal-Wallis H tests, and are shown as median (25^th^, 75^th^ percentile).

### Impact of HOMA-IR increases on the development of type 2 diabetes in individuals with IIS

At follow-up examination, 74 individuals (22 individuals in the non-IIS group and 52 individuals in the IIS group) developed type 2 diabetes. Because the youngest of these individuals was 40 years old at baseline examination, all incident cases were assumed to be type 2 diabetes. Table 2 shows the ORs for the development of type 2 diabetes among the eight groups. The multivariable-adjusted ORs (95% CIs) for the development of type 2 diabetes in the non-IIS with a decrease, non-IIS with a moderate increase, non-IIS with a large increase, IIS with a decrease, IIS with no change/small increase, IIS with a moderate increase, and IIS with a large increase groups, relative to the non-IIS with no change/small increase group were 0.23 (0.04, 1.11), 1.22 (0.26, 5.72), 2.01 (0.70, 6.46), 1.37 (0.32, 4.28), 3.60 (0.83, 15.57), 5.24 (1.34, 20.52), and 7.01 (1.75, 24.18), respectively. We observed similar results when we added the eight individuals who received medical treatment for diabetes between the baseline and follow-up examinations.

**Table 2:** ORs for the development of type 2 diabetes according to baseline IIS status and category of ΔHOMA-IR.

	Non-IIS				IIS			
	Category of ΔHOMA-IR				Category of ΔHOMA-IR			
	Decrease (*n* = 817)	No change/Small increase (*n* = 236)	Moderate increase (*n* = 227)	Large increase (*n* = 260)	Decrease (*n* = 300)	No change/Small increase (*n* = 128)	Moderate increase (*n* = 137)	Large increase (*n* = 104)
Incident cases, n (%)	5 (0.6)	4 (1.7)	4 (1.8)	9 (3.5)	17 (5.7)	6 (4.7)	12 (8.8)	17 (16.3)
OR (95% CI)								
Model 1	0.32 (0.08, 1.24)	1.0	1.11 (0.26, 4.64)	2.35 (0.90, 7.05)	3.51 (0.86, 8.50)	3.11 (0.82, 11.76)	5.71 (1.69, 19.27)	8.84 (2.70, 28.95)
Model 2	0.23 (0.04, 1.11)	1.0	1.22 (0.26, 5.72)	2.01 (0.70, 6.46)	1.37 (0.32, 4.28)	3.60 (0.83, 15.57)	5.24 (1.34, 20.52)	7.01 (1.75, 24.18)

IIS, impaired insulin secretion; HOMA-IR, homeostasis model assessment of insulin resistance; OR, odds ratio; CI, confidence interval.

Non-IIS, insulinogenic index >51.7 pmol/mmol (40.0 µU/mg); IIS, insulinogenic index ≤51.7 pmol/mmol (40.0 µU/mg).

A decrease group consisted of individuals with a decrease (<0) in HOMA-IR, whereas those with no change or an increase in HOMA-IR were divided into tertiles; a no change/small increase group, tertile 1 (0.00–0.16); a moderate increase group, tertile 2 (0.17–0.40); and a large increase group, tertile 3 (≥0.41).

Model 1 was adjusted for age, sex, and follow-up years (3 or 4 years).

Model 2 was adjusted for all factors in model 1 plus family history of diabetes (yes or no), current smoking (yes or no), alcohol consumption (0 g/week, 1–139 g/week or ≥140 g/week), exercise (0 min/week, 1–119 min/week or ≥120 min/week), baseline HOMA-IR, fasting plasma glucose, and 2 h post-load plasma glucose.

Table S1 in File S1 shows ORs for the development of type 2 diabetes according to baseline IIS status and another category of ΔHOMA-IR (decrease, ≤−0.20; stable, ±0.19; moderate increase, 0.20–0.39; and large increase, ≥0.40). The multivariable-adjusted ORs (95% CIs) for the development of type 2 diabetes in the IIS with a moderate increase and IIS with a large increase groups, relative to the non-IIS with stable HOMA-IR group were 5.88 (1.51, 19.65) and 8.92 (2.66, 29.83), respectively.

**Figure 1:**
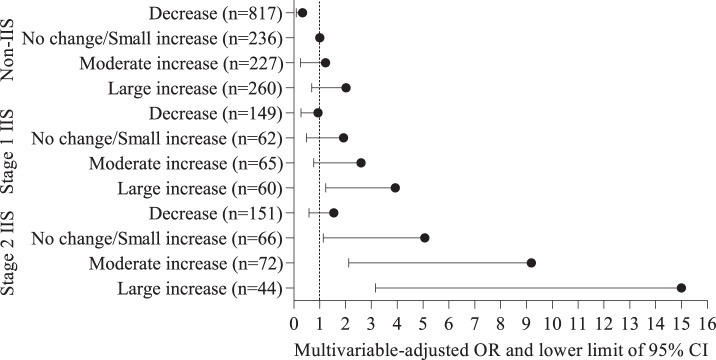
ORs for the development of type 2 diabetes according to baseline IIS status (non-IIS, stage 1 IIS, and stage 2 IIS) and category of ΔHOMA-IR. IIS, impaired insulin secretion; HOMA-IR, homeostasis model assessment of insulin resistance; OR, odds ratio; CI, confidence interval. Non-IIS, insulinogenic index >51.7 pmol/mmol (40.0 µU/mg); stage 1 IIS, insulinogenic index 35.5–51.7 pmol/mmol (27.5–40.0 µU/mg); stage 2 IIS, insulinogenic index 2.8–35.4 pmol/mmol (2.2–27.4 µU/mg). A decrease group consisted of individuals with a decrease (<0) in HOMA-IR, whereas those with no change or an increase in HOMA-IR were divided into tertiles; a no change/small increase group, tertile 1 (0.00–0.16); a moderate increase group, tertile 2 (0.17–0.40); and a large increase group, tertile 3 (≥0.41). Data were adjusted for age, sex, follow-up years (3 or 4 years), family history of diabetes (yes or no), current smoking (yes or no), alcohol consumption (0 g/week, 1–139 g/week or ≥140 g/week), exercise (0 min/week, 1–119 min/week or ≥120 min/week), baseline HOMA-IR, fasting plasma glucose, and 2 h post-load plasma glucose.

Figure 1 shows ORs for the development of type 2 diabetes among 12 groups according to the combination of baseline IIS status (non-IIS, stage 1 IIS, and stage 2 IIS) and category of ΔHOMA-IR (decrease, no change/small increase, moderate increase, and large increase). The multivariable-adjusted ORs (95% CIs) for the development of type 2 diabetes in the stage 1 IIS with a large increase (age- and sex-adjusted mean ΔHOMA-IR: 0.70), stage 2 IIS with no change/small increase (age- and sex-adjusted mean ΔHOMA-IR: 0.10), stage 2 IIS with a moderate increase (age- and sex-adjusted mean ΔHOMA-IR: 0.28), and stage 2 IIS with a large increase (age- and sex-adjusted mean ΔHOMA-IR: 0.73) groups, relative to the non-IIS with no change/small increase group were 3.93 (1.23, 12.54), 5.07 (1.14, 22.76), 9.19 (2.12, 39.79), and 15.00 (3.16, 62.00), respectively.

### Risk factors for an increase in HOMA-IR

Table 3 shows ORs for an increase in HOMA-IR according to major risk factors for diabetes in the non-IIS and IIS groups. ΔBMI and Δwaist circumference were significant risk factors for moderate and large increases in HOMA-IR in both the non-IIS and IIS groups. Baseline BMI was a significant risk factor for a large increase in the non-IIS group.

**Table 3:** ORs for an increase in HOMA-IR according to major risk factors for diabetes in the non-IIS and IIS groups.

Variable	Adjusted OR (95% CI)	
	For a moderate increase in HOMA-IR	For a large increase in HOMA-IR
Non-IIS		
Age (per year)	1.01 (0.99–1.03)	0.99 (0.97–1.01)
Sex (men/women)	1.20 (0.88–1.64)	0.99 (0.73–1.34)
Family history of diabetes (yes/no)	1.12 (0.76–1.64)	1.28 (0.90–1.84)
Baseline BMI (per kg/m^2^)	0.93 (0.84–1.02)	1.10 (1.02, 1.20)
Baseline waist circumference (per cm)	1.00 (0.96, 1.03)	1.02 (0.98, 1.05)
ΔBMI (per kg/m^2^)	1.69 (1.40, 2.05)	1.92 (1.60–2.30)
Δwaist circumference (per cm)	1.04 (1.01, 1.09)	1.05 (1.02, 1.10)
IIS		
Age (per year)	1.00 (0.97, 1.03)	0.99 (0.96, 1.02)
Sex (men/women)	1.37 (0.87–2.13)	0.69 (0.42–1.12)
Family history of diabetes (yes/no)	0.99 (0.61–1.61)	0.97 (0.56–1.69)
Baseline BMI (per kg/m^2^)	0.88 (0.75, 1.03)	1.04 (0.89–1.23)
Baseline waist circumference (per cm)	1.04 (0.96, 1.15)	1.06 (0.99, 1.13)
ΔBMI (per kg/m^2^)	1.23 (1.03, 1.59)	1.66 (1.25, 2.20)
Δwaist circumference (per cm)	1.12 (1.04, 1.20)	1.10 (1.02, 1.18)

IIS, impaired insulin secretion; HOMA-IR, homeostasis model assessment of insulin resistance; OR, odds ratio; CI, confidence interval; BMI, body mass index.

Non-IIS, insulinogenic index >51.7 pmol/mmol (40.0 µU/mg); IIS, insulinogenic index ≤51.7 pmol/mmol (40.0 µU/mg).

Moderate increase, 0.17–0.40 increase in HOMA-IR; large increase, ≥0.41 increase in HOMA-IR.

Δ = follow-up examination minus baseline examination.

Data were adjusted for all variables in table plus follow-up years (3 or 4 years), current smoking (yes or no), alcohol consumption (0 g/week, 1–139 g/week or ≥140 g/week), exercise (0 min/week, 1–119 min/week or ≥120 min/week), baseline HOMA-IR, fasting plasma glucose, and 2 h post-load plasma glucose.

## Discussion

This community-based cohort study clearly shows the relationship of HOMA-IR increases on the development of type 2 diabetes in Japanese individuals with IIS. Our main finding was that, after adjusting for confounding factors including baseline HOMA-IR values, the ORs for type 2 diabetes were 5.24 in the IIS with a moderate increase group and 7.01 in the IIS with a large increase group when compared with the non-IIS with no change/small increase group. Additionally, when IIS was classified into stage 1 (insulinogenic index 35.5–51.7 pmol/mmol) and stage 2 (insulinogenic index 2.8–35.4 pmol/mmol), the ORs for type 2 diabetes were 3.93 in the stage 1 IIS with a large increase group, 5.07 in the stage 2 IIS with no change/small increase group, 9.19 in the stage 2 IIS with a moderate increase group, and 15.00 in the stage 2 IIS with a large increase group, relative to the non-IIS with no change/small increase group.

Our findings indicate that moderate and large increases in HOMA-IR had a strong impact on the development of type 2 diabetes among individuals with IIS in this Japanese population, regardless of baseline HOMA-IR, FPG, and 2 h PG. On the other hand, moderate and large increases in HOMA-IR did not increase the risk for type 2 diabetes in those without IIS. Each mean HOMA-IR increment in the IIS with a moderate increase group (0.27) and a large increase group (0.73) was similar to that in the non-IIS with a moderate increase group (0.28) and a large increase group (0.83). Therefore, it is thought that those with IIS cannot compensate for moderate and large increases in HOMA-IR (even if the increase is within the normal range) because of their low insulin-secreting ability. When we calculated the value corresponding to the maximum Youden index (sensitivity + specificity − 1) using receiver operating characteristic curves analysis, the optimal cut-off point for ΔHOMA-IR was 0.24 in the IIS group (data not shown). It is necessary for those with IIS to be more careful about increases of IR. On the other hand, it is thought that non-IIS individuals with moderate and large increases in HOMA-IR might have delayed onset of diabetes compared with those with IIS. It has been reported that the development of diabetes following the development of IR can take several years [13], [14]. In our study, median insulinogenic indices at follow-up examination were 81.0 pmol/mmol in the non-IIS with a moderate increase group and 106.4 pmol/mmol in the non-IIS with a large increase group (data not shown). Therefore, it is thought that non-IIS individuals with moderate or large increases in HOMA-IR could compensate for the increases in HOMA-IR in this short-term follow-up study. In the 260 non-IIS individuals with large increases, the proportion of those who had pre-diabetes increased from 18% at baseline examination to 32% at follow-up examination (data not shown). Therefore, non-IIS individuals, especially those with large increases in HOMA-IR, are still at risk for type 2 diabetes.

The association between moderate and large increases in HOMA-IR and the development of type 2 diabetes was strengthened in the stage 2 IIS group, i.e., severe IIS. The impact of an increase in HOMA-IR on the development of type 2 diabetes in Japanese individuals would differ depending on the degree of insulin-secreting ability. Therefore, both the assessment of insulin-secreting ability in individuals without diabetes and the stricter prevention of IR increases in those with low insulin-secreting ability will be necessary for the prevention of type 2 diabetes in Japanese individuals. Regarding the prevention of IR increases, weight gain over a short period of time was associated with increased IR development regardless of baseline BMI status [15]. In the present study, ΔBMI and Δwaist circumference were risk factors for moderate and large increases in HOMA-IR in both the non-IIS and IIS groups. Therefore, the control of weight and waist circumference will be important in preventing IR increases in individuals with low insulin-secreting ability.

The risk for type 2 diabetes increased in the stage 2 IIS group (i.e., severe IIS) with no change/small increase in HOMA-IR. Insulin secretion in Japanese individuals is reported to be less than half that of whites [16], [17], and most Japanese patients with type 2 diabetes do not have IR [18]. Additionally, studies in Sweden and those conducted in Pima Native Americans have shown that the acute insulin secretory response to intravenous glucose is an independent predictor of the development of diabetes [19]–[22]. Similarly, a low incremental 30 min insulin response during the OGTT was found to be a predictor of the development of diabetes in Mexican Americans, independent of obesity and fasting insulin concentrations [23]. Therefore, prevention and control of decreases in insulin-secreting ability are important to prevent type 2 diabetes in Japanese individuals. Genetic factors and acquired abnormalities largely secondary to unhealthy lifestyles can affect insulin secretion. Regarding genetic factors, the *KCNQ1* and *KCNJ15* variants have been reported to affect the development of type 2 diabetes by impairing beta cell function [24]–[27]. Regarding acquired abnormalities largely secondary to unhealthy lifestyles, we recently reported that cigarette smoking is a modifiable risk factor for the development of IIS [28]. However, identification of the modifiable risk factors for IIS is not enough and additional studies are needed.

Of the 300 individuals with IIS for whom we recorded a decrease in HOMA-IR, 17 (5.7%) developed type 2 diabetes despite the decrease in their HOMA-IR. At baseline, 12 (70.6%) of these 17 individuals had pre-diabetes, and 6 (35.3%) had a family history of diabetes (data not shown). Additionally, the mean HOMA-IR of these 17 individuals was 1.52, and the median insulinogenic index was 22.4 pmol/mmol (data not shown). It is thought that these 17 individuals with IIS with a decrease in HOMA-IR developed type 2 diabetes as a result of their baseline status, especially the high proportion of pre-diabetes, HOMA-IR value, and low insulin secretion.

The strengths of the present study include its community-based cohort, which consisted of residents of many cities throughout Nagano prefecture. Furthermore, we screened all participants for type 2 diabetes by the 75 g OGTT. The 12 h overnight fast before the OGTT was managed by hospitalising participants the day before the test. This study, however, also had several limitations. First, the estimates of insulin secretion and IR were made using calculations based on the OGTT, not by the ‘gold standard’ test, i.e., the glucose-clamp technique. However, the glucose-clamp technique is not feasible in large-scale epidemiological studies, and we believe that proxy measures are reliable in large datasets. Second, estimation of IR by calculating the HOMA-IR, which primarily reflects hepatic IR [29], was another potential limitation. Third, in several cases, the 95% CIs calculated were very wide because of small sample sizes in some groups. Fourth, there may have been a possibility of selection bias, as the participants in this study were individuals who underwent routine comprehensive medical check-ups. Although these check-ups are generally expensive in Japan, those at Saku Central Hospital are relatively inexpensive or free, because administrations and employers subsidise their costs. Therefore, many community residents undergo these examinations, and the rates of diabetes and overweight/obesity in our cohort were similar to those observed in the general Japanese population [30]. Finally, individuals who did not undergo a comprehensive medical check-up between April 2009 and March 2011 were excluded from our analysis. However, baseline characteristics such as age, BMI, waist circumference, HOMA-IR, insulinogenic index, and FPG did not differ significantly between individuals who were eligible for our analysis and those who did not undergo a comprehensive medical check-up between April 2009 and March 2011 (data not shown).

In conclusion, moderate and large increases in HOMA-IR had a strong impact on the development of type 2 diabetes regardless of baseline HOMA-IR values among individuals with IIS in this Japanese population. The National Nutrition Survey, a nationwide survey in Japan, has reported an increase in the number of overweight Japanese men during the past 20–30 years because of changes in eating habits and decline in physical activity [31], [32]. Consequently, the increase in the number of overweight Japanese individuals who have low insulin-secreting ability is likely to be reflected by further increases in the number of Japanese individuals with type 2 diabetes.

## Supporting Information

File S1 **Combined supporting information file. Table S1. ORs for the development of type 2 diabetes according to baseline IIS status and another category of ΔHOMA-IR (decrease, ≤−0.20; stable, ±0.19; moderate increase, 0.20–0.39; and large increase, ≥0.40).** Note: IIS, impaired insulin secretion; HOMA-IR, homeostasis model assessment of insulin resistance; OR, odds ratio; CI, confidence interval. Non-IIS, insulinogenic index >51.7 pmol/mmol (40.0 µU/mg); IIS, insulinogenic index ≤51.7 pmol/mmol (40.0 µU/mg). Δ = follow-up examination minus baseline examination. ΔHOMA-IR was analyzed by analysis of covariance with adjustments for age and sex, and is shown as age- and sex-adjusted mean (95% confidence interval). Model 1 was adjusted for age, sex, and follow-up years (3 or 4 years). Model 2 was adjusted for all factors in model 1 plus family history of diabetes (yes or no), current smoking (yes or no), alcohol consumption (0 g/week, 1-139 g/week or ≥140 g/week), exercise (0 min/week, 1-119 min/week or ≥120 min/week), baseline HOMA-IR, fasting plasma glucose, and 2 h post-load plasma glucose. **Figure S1. Definition of IIS and category of HOMA-IR change.** Note: IIS, impaired insulin secretion; HOMA-IR, homeostasis model assessment of insulin resistance. **Figure S2. HOMA-IR (A-1 and A-2), BMI (B-1 and B-2), and waist circumference (C-1 and C-2) at baseline and follow-up examinations among individuals who developed type 2 diabetes (i.e., incident cases) and individuals who maintained normal glucose regulation (i.e., controls) in the non-IIS and IIS groups.** Note: IIS, impaired insulin secretion; HOMA-IR, homeostasis model assessment of insulin resistance; BMI, body mass index. Data are shown as age- and sex-adjusted means (95% confidence intervals). Incident cases in the non-IIS group: *n* = 22; controls in the non-IIS group: *n* = 1,518; incident cases in the IIS group: *n* = 52; controls in the IIS group: *n* = 617.
(PDF)
