# Correction: Increase in Homeostasis Model Assessment of Insulin Resistance (HOMA-IR) Had a Strong Impact on the Development of Type 2 Diabetes in Japanese Individuals with Impaired Insulin Secretion: The Saku Study

**DOI:** 10.1371/journal.pone.0116584

**Published:** 2014-12-23

**Authors:** The PLOS ONE Staff

There is an error in Table 1. The publisher apologizes for this error. Please view the corrected Table 1 here.

**Table 1:** Characteristics according to baseline IIS status and category of ΔHOMA-IR.

Characteristic	Non-IIS				IIS				*p* value
	Category of ΔHOMA-IR				Category of ΔHOMA-IR				
	Decrease (*n* = 817)	No change/Small increase (*n* = 236)	Moderate increase (*n* = 227)	Large increase (*n* = 260)	Decrease (*n* = 300)	No change/Small increase (*n* = 128)	Moderate increase (*n* = 137)	Large increase (*n* = 104)	
Insulinogenic index (pmol/mmol)	103.4 (74.1, 168.6)	93.6 (69.0, 146.2)	92.0 (65.1, 135.7)	107.3 (74.3, 165.5)	35.3 (23.0, 44.2)	35.1 (25.7, 43.3)	34.8 (26.8, 43.7)	37.8 (27.0, 45.5)	
ΔHOMA-IR	−0.47 (−0.50, −0.44)	0.08 (0.04, 0.12)	0.28 (0.23, 0.34)	0.83 (0.77, 0.88)	−0.36 (−0.40, −0.31)	0.08 (0.04, 0.12)	0.27 (0.20, 0.34)	0.73 (0.64, 0.81)	
Baseline HOMA-IR	1.47 (1.42, 1.52)	0.93 (0.83, 1.02)	0.90 (0.80, 0.99)	1.26 (1.17, 1.35)	1.10 (1.01, 1.18)	0.70 (0.58, 0.83)	0.66 (0.54, 0.78)	0.89 (0.75, 1.04)	<0.001
Age (years)	55.4 (54.9, 55.9)	55.5 (54.6, 56.5)	55.4 (54.4, 56.4)	54.3 (53.4, 55.2)	56.5 (55.7, 57.4)	56.1 (54.8, 57.5)	56.2 (54.9, 57.5)	55.2 (53.7, 56.7)	0.047
Men, n (%)	412 (50)	132 (56)	119 (52)	146 (56)	177 (59)	82 (64)	94 (69)	63 (61)	0.001
Family history of diabetes, n (%)	146 (18)	39 (17)	41 (18)	55 (21)	60 (20)	30 (23)	29 (21)	25 (24)	0.499
Current smoker, n (%)	110 (14)	34 (14)	36 (16)	46 (18)	64 (21)	34 (27)	39 (29)	33 (32)	<0.001
Alcohol consumption (ethanol), n (%)									0.002
0 g/week	304 (37)	87 (37)	86 (38)	88 (34)	86 (29)	36 (28)	34 (25)	36 (35)	
1-139 g/week	344 (42)	86 (36)	100 (44)	116 (45)	128 (43)	56 (44)	54 (39)	45 (43)	
≥140 g/week	169 (21)	63 (27)	41 (18)	56 (22)	86 (29)	36 (28)	49 (36)	23 (22)	
Exercise, n (%)									0.358
0 min/week	383 (47)	115 (49)	101 (45)	133 (51)	134 (45)	58 (45)	64 (47)	49 (47)	
1-119 min/week	283 (35)	68 (29)	69 (30)	80 (31)	101 (34)	46 (36)	40 (29)	27 (26)	
≥120 min/week	151 (19)	53 (23)	57 (25)	47 (18)	65 (22)	24 (19)	33 (24)	28 (27)	
BMI (kg/m^2^)	23.5 (23.3, 23.7)	22.8 (22.4, 23.2)	22.5 (22.1, 22.9)	24.2 (23.9, 24.6)	22.6 (22.3, 22.9)	22.1 (21.6, 22.5)	22.1 (21.6, 22.5)	23.6 (23.1, 24.1)	<0.001
