# Supplementary material for: Increase in Homeostasis Model Assessment of Insulin Resistance (HOMA-IR) Had a Strong Impact on the Development of Type 2 Diabetes in Japanese Individuals with Impaired Insulin Secretion: The Saku Study
Source: PLoS One. 2014 Aug 28;9(8):e105827. doi: 10.1371/journal.pone.0105827 (PMC4148342; doi:10.1371/journal.pone.0105827)
Supplement: File S1 — Combined supporting information file. Table S1. ORs for the development of type 2 diabetes according to baseline IIS status and another category of ΔHOMA-IR (decrease, ≤−0.20; stable, ±0.19; moderate increase, 0.20–0.39; and large increase, ≥0.40). Note: IIS, impaired insulin secretion; HOMA-IR, homeostasis model assessment of insulin resistance; OR, odds ratio; CI, confidence interval. Non-IIS, insulinogenic index >51.7 pmol/mmol (40.0 µU/mg); IIS, insulinogenic index ≤51.7 pmol/mmol (40.0 µU/mg). Δ = follow-up examination minus baseline examination. ΔHOMA-IR was analyzed by analysis of covariance with adjustments for age and sex, and is shown as age- and sex-adjusted mean (95% confidence interval). Model 1 was adjusted for age, sex, and follow-up years (3 or 4 years). Model 2 was adjusted for all factors in model 1 plus family history of diabetes (yes or no), current smoking (yes or no), alcohol consumption (0 g/week, 1-139 g/week or ≥140 g/week), exercise (0 min/week, 1-119 min/week or ≥120 min/week), baseline HOMA-IR, fasting plasma glucose, and 2 h post-load plasma glucose. Figure S1. Definition of IIS and category of HOMA-IR change. Note: IIS, impaired insulin secretion; HOMA-IR, homeostasis model assessment of insulin resistance. Figure S2. HOMA-IR (A-1 and A-2), BMI (B-1 and B-2), and waist circumference (C-1 and C-2) at baseline and follow-up examinations among individuals who developed type 2 diabetes (i.e., incident cases) and individuals who maintained normal glucose regulation (i.e., controls) in the non-IIS and IIS groups. Note: IIS, impaired insulin secretion; HOMA-IR, homeostasis model assessment of insulin resistance; BMI, body mass index. Data are shown as age- and sex-adjusted means (95% confidence intervals). Incident cases in the non-IIS group: n = 22; controls in the non-IIS group: n = 1,518; incident cases in the IIS group: n = 52; controls in the IIS group: n = 617. (PDF) [file pone.0105827.s001.pdf]

Supporting Information File S1

Table S1. ORs for the development of type 2 diabetes according to baseline IIS status and another category of ΔHOMA-IR (decrease, ≤−0.20; stable, ±0.19; moderate increase, 0.20-0.39; and large increase, ≥0.40)

	**Non-IIS**				**IIS**			
	**Category of ΔHOMA-IR**				**Category of ΔHOMA-IR**			
**Variable**	**Decrease, ≤−0.20 (*n*=567)**	**Stable, ±0.19 (*n*=519)**	**Moderate increase, 0.20-0.39 (*n*=187)**	**Large increase, ≥0.40 (*n*=267)**	**Decrease, ≤−0.20 (*n*=163)**	**Stable, ±0.19 (*n*=298)**	**Moderate increase, 0.20-0.39 (*n*=100)**	**Large increase, ≥0.40 (*n*=108)**
ΔHOMA-IR	−0.63 (−0.66, −0.60)	0.00 (−0.03, 0.03)	0.29 (0.24, 0.35)	0.81 (0.77, 0.86)	−0.57 (−0.63, −0.51)	0.01 (−0.03, 0.05)	0.28 (0.23, 0.34)	0.71 (0.64, 0.79)
Incident cases, n (%)	4 (0.7)	5 (1.0)	3 (1.6)	10 (3.7)	14 (8.6)	12 (4.0)	9 (9.0)	17 (15.7)
OR (95% CI)								
Model 1	0.65 (0.17, 2.47)	1.0	1.63 (0.38, 7.06)	3.90 (1.27, 11.91)	7.37 (2.50, 21.75)	4.00 (1.03, 14.41)	7.91 (2.30, 23.83)	14.29 (4.84, 42.15)
Model 2	0.27 (0.06, 1.16)	1.0	1.53 (0.33, 7.22)	2.99 (0.88, 10.17)	1.97 (0.58, 6.62)	3.68 (0.89, 12.57)	5.88 (1.51, 19.65)	8.92 (2.66, 29.83)

IIS, impaired insulin secretion; HOMA-IR, homeostasis model assessment of insulin resistance; OR, odds ratio; CI, confidence interval.

Non-IIS, insulinogenic index >51.7 pmol/mmol (40.0 μU/mg); IIS, insulinogenic index ≤51.7 pmol/mmol (40.0 μU/mg).

Δ=follow-up examination minus baseline examination.

ΔHOMA-IR was analyzed by analysis of covariance with adjustments for age and sex, and is shown as age- and sex-adjusted mean (95% confidence interval).

Model 1 was adjusted for age, sex, and follow-up years (3 or 4 years).

Model 2 was adjusted for all factors in model 1 plus family history of diabetes (yes or no), current smoking (yes or no), alcohol consumption (0 g/week, 1-139 g/week or ≥140 g/week), exercise (0 min/week, 1-119 min/week or ≥120 min/week), baseline HOMA-IR, fasting plasma glucose, and 2 h post-load plasma glucose.

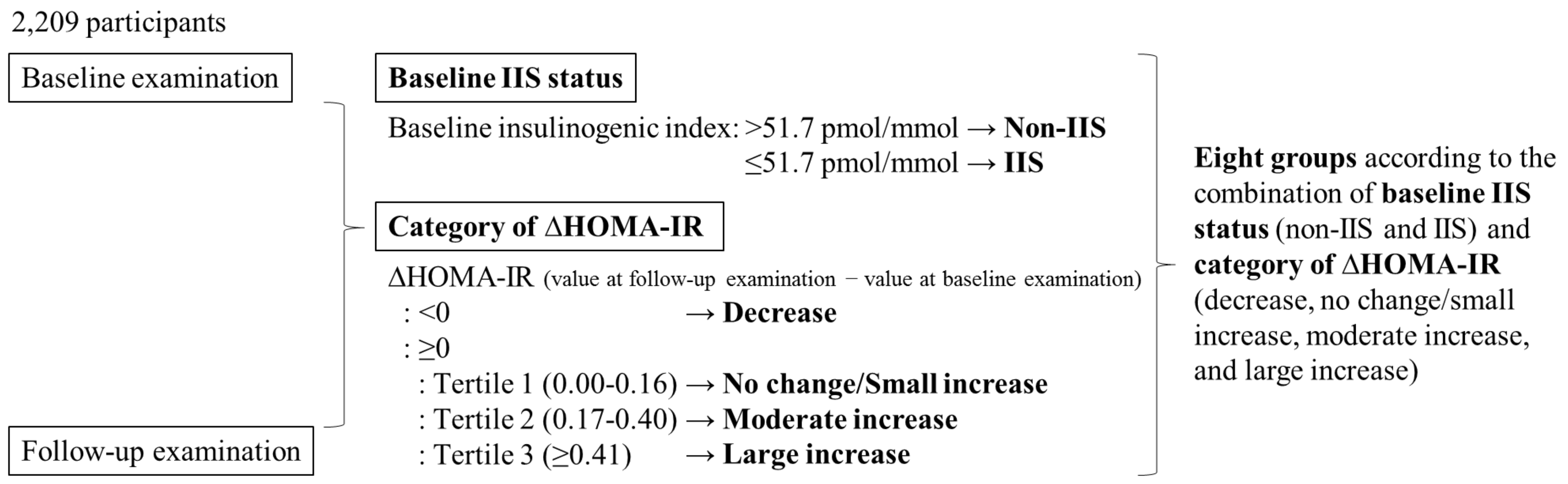

Figure S1. Definition of IIS and category of HOMA-IR change

IIS, impaired insulin secretion; HOMA-IR, homeostasis model assessment of insulin resistance.

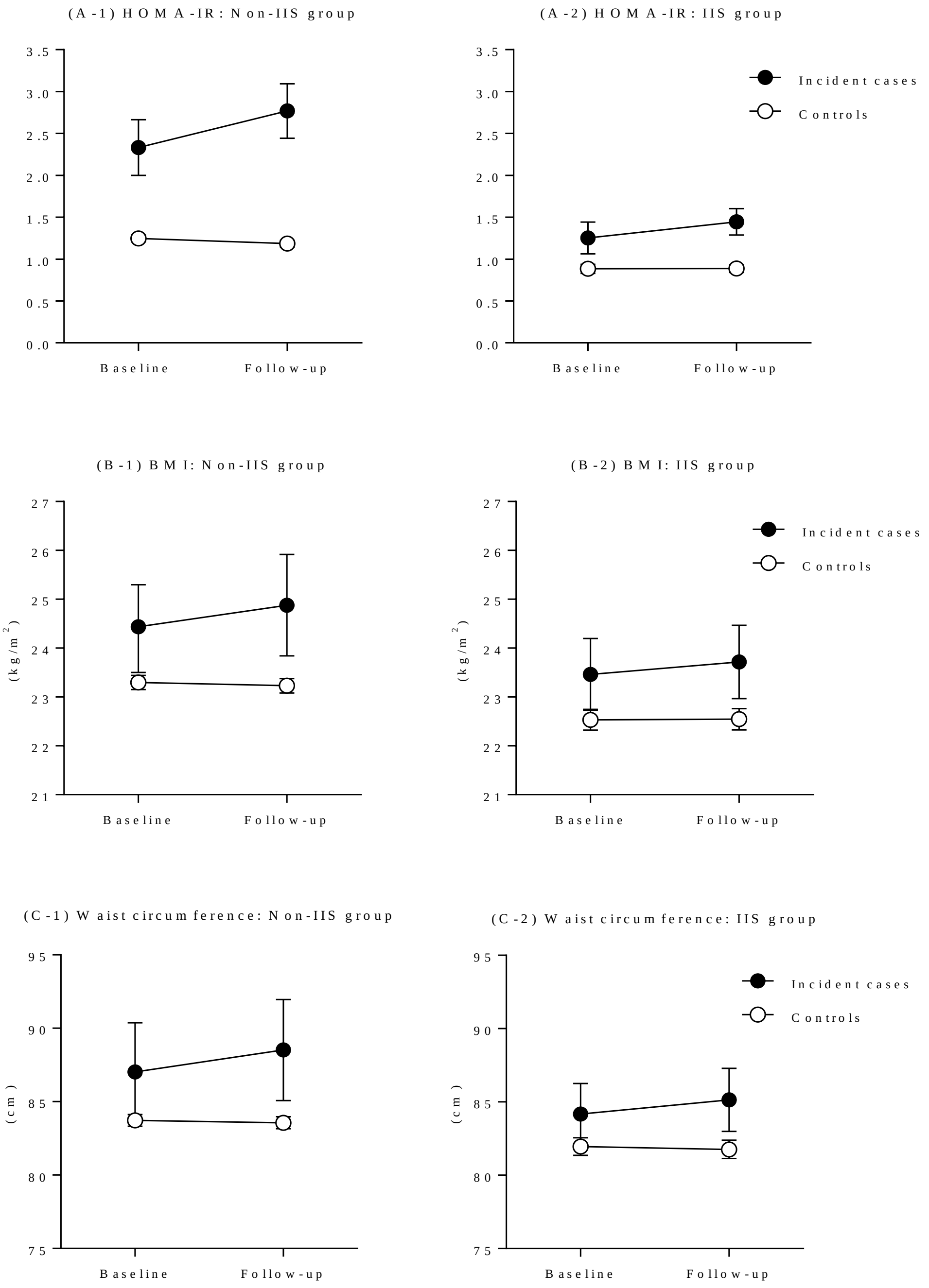

Figure S2. HOMA-IR (A-1 and A-2), BMI (B-1 and B-2), and waist circumference (C-1 and C-2) at baseline and follow-up examinations among individuals who developed type 2 diabetes (i.e., incident cases) and individuals who maintained normal glucose regulation (i.e., controls) in the non-IIS and IIS groups.

IIS, impaired insulin secretion; HOMA-IR, homeostasis model assessment of insulin resistance; BMI, body mass index.

Data are shown as age- and sex-adjusted means (95% confidence intervals).

Incident cases in the non-IIS group: *n*=22; controls in the non-IIS group: *n*=1,518; incident cases in the IIS group: *n*=52; controls in the IIS group: *n*=617.
